# Additional Analgesia for Central Venous Catheter Insertion: A Placebo Controlled Randomized Trial of Dexmedetomidine and Fentanyl

**DOI:** 10.1155/2016/9062658

**Published:** 2016-04-21

**Authors:** Aloka Samantaray, Mangu Hanumantha Rao, Chitta Ranjan Sahu

**Affiliations:** ^1^Department of Anaesthesiology and Critical Care, Sri Venkateswara Institute of Medical Sciences, Tirupati, Andhra Pradesh 517507, India; ^2^Department of Orthopaedics, Balaji Institute of Surgery, Research and Rehabilitation for the Disabled, Tirupati, Andhra Pradesh 517507, India

## Abstract

We aimed to show that a single preprocedural dose of either dexmedetomidine or fentanyl reduces procedural pain and discomfort and provides clinically acceptable sedation. In this prospective, double-blind study, sixty patients scheduled for elective surgery and requiring planned central venous catheter insertion were randomized to receive dexmedetomidine (1 *μ*g/kg), fentanyl (1 *μ*g/kg), or 0.9% normal saline intravenously over ten minutes followed by local anesthetic field infiltration before attempting central venous catheterization. The primary outcome measures are assessment and analysis of pain, discomfort, and sedation level before, during, and after the central venous catheter insertion at five time points. The median (IQR) pain score is worst for normal saline group at local anaesthetic injection [6 (4–6.7)] which was significantly attenuated by addition of fentanyl [3 (2–4)] and dexmedetomidine [4 (3–5)] in the immediate postprocedural period (*P* = 0.001). However, the procedure related discomfort was significantly lower in dexmedetomidine group compared to fentanyl group in the first 10 min of procedure after local anaesthetic Injection (*P* = 0.001). Fentanyl is more analgesically efficient for central venous catheter insertion along with local anaesthetic injection. However, dexmedetomidine has the potential to be superior to fentanyl and placebo in terms of providing comfort to the patients during the procedure.

## 1. Introduction

Hospitals that are hard pressed to operate more number of cases per day usually carry out procedures like epidural catheter insertion or central venous catheter insertion (CVCI) in the holding area outside the operation theatre (OT) for the second case, while the first case of the day is still inside the operation theatre. Though this strategy reduces the surgical readiness time for the second case [1], the conscious patient has to undergo pain and discomfort of the procedures like CVCI. The positional requirement for CVCI like making the patient lay perfectly still in Trendelenburg position with a partially flexed neck turned to one side and extended head is a source of discomfort. Most clinicians including the author believe that the first needle prick of local anaesthesia (LA) will give maximum pain stimulus compared to subsequent procedural steps like using the locator needle and anchoring the central venous catheter (CVC) to skin. The moderately painful and severely uncomfortable condition associated with CVCI may further escalate their anxiety if adequate analgesia and patient comfort are not ensured during this presurgical period.

A multicenter American study observed that 51.5% of patients had an increase in pain intensity immediately after the procedure (CVCI) over the baseline preprocedural pain [2]. Two single-center studies from India have shown that injection of local anesthetics causes the worst pain and both dexmedetomidine and fentanyl can attenuate the pain and discomfort of CVCI to variable degree and the authors suggested a cautious approach while selecting a particular drug for routine use owing to their reported side effects [3, 4]. There are no further studies available comparing the potential of dexmedetomidine and fentanyl in conjunction with LA field infiltration in reducing the pain and discomfort associated with CVCI.

The purpose of this prospective, randomized, double-blind clinical trial is to evaluate objectively the effects of addition a single dose of either fentanyl or dexmedetomidine to conventional subcutaneous LA infiltration analgesia on perceived pain and discomfort during CVCI.

## 2. Materials and Methods

The study protocol was approved by the Institute's Ethics Committee (IEC number: 350) on 30th January 2014 and written informed consent was obtained from each participant. The study protocol was registered with Clinical Trials Registry-India (CTRI/2014/02/004397). The study was conducted in a randomized, double-blinded, placebo-controlled manner and conformed to the CONSORT guidelines. The study protocol conforms to the provisions of the Declaration of Helsinki in 1995 (as revised in Seoul 2008).

Sixty consecutive adult patients of American Society of Anesthesiologists physical status 1 and 2 scheduled for elective surgery under general anaesthesia and requiring central venous access with planned placement in the internal jugular vein (IJV) as a part of normal care were enrolled in the study after obtaining due consent. Patients were included in the study if they were with 18–65 years, awake, alert, and oriented and their medical condition was stable enough to allow them to understand and use a verbal numeric rating pain scale (VNRPS) and systemic analgesics had not been administered for at least four hours before the procedure. All the patients received their routine premedication like alprazolam (0.5 mg, oral) night before surgery and on the day of surgery. Patients were excluded from the study if they were on any medication meant for controlling heart rate (HR) and blood pressure, had a known adverse reaction to the study drugs, had past history of undergoing any surgery with or without anaesthesia in the preceding one year, and were not willing to participate in the study.

All the study participants were called to the holding area adjacent to OT 90 min before the scheduled surgery and were allocated randomly to one of the three groups using a computer-generated random number table and a sealed opaque envelope technique. An anesthetist, who was not one of the observers, prepared syringes containing either dexmedetomidine 1 *μ*g/kg, fentanyl 1 *μ*g/kg, or 0.9% saline (placebo group), all made to a total volume of 10 mL. All the solutions were labeled “study drug” and coded to maintain the double-blind nature of the study. The study drugs were infused to the patients as per their group allocation over 10 min using a syringe infusion pump.

The procedure for CVCI commenced at the end of infusion of the study drugs. Each patient was positioned supine with a rolled up towel placed between the scapulae to extend the head and the patient was given 10–15° Trendelenburg position with the neck turned to the opposite side. A standard subcutaneous infiltration of 5 mL of 2% lignocaine was made after confirming the anatomical landmark of the target jugular vein. A physician blinded to the study drug injected 3 mL of the LA solution over 10 to 15 seconds through a 25-gauge needle at the apex of the triangle formed by the lateral and medial head of sternocleidomastoid muscle using anterior approach [5]; an additional 2 mL of LA in divided doses (1 mL on either side) was injected above and on either side of the vein for anchoring stitches by just repositioning the needle. Each patient received 7 French CVC via a nontunneled approach. Ultrasonography was not used in any of the study subjects as it was not available at our institution at the time of carrying out the study and moreover Indian Society of Anesthesiologists does not mandate use of ultrasound for central venous catheterization, unlike in academic medical centers in North America, Europe, and even many countries in Asia.

Scores for discomfort, pain, and sedation were recorded at rest by an anesthesia resident at five time points: BL, before starting study drug infusion; LAI, after initial LA injection; PP3, immediately after the CVCI, the patient was asked to report the peak pain experienced during the procedure and rate the discomfort during the procedure; PP10, 10 min after completion of the procedure; and PP60, 60 min after completion of the procedure. Patients were also closely monitored for any adverse effects of study drugs like respiratory depression, nausea, or vomiting.

Pain and discomfort are assessed using an 11-point VNRPS or* verbal numeric rating discomfort/distress scale [6]* (VNRDS) from 0 to 10, where “0” represents no pain or no discomfort and 10 represents worst imaginable pain or extreme discomfort, respectively. The scale was explained to each patient by the investigator while counseling the patient regarding the need for central venous access before the start of infusion of study drugs. Sedation was assessed on a 6-point modified Observer's Assessment of Alertness/Sedation (OAA/S) Scale with 0 indicating no response and 6 indicating that the patient is agitated [7]. If OAA/S score was 0 or 1 (patient unarousable), VNRPS and VNRDS were counted as 0 (no pain and no discomfort).

Heart rate (HR), systolic blood pressure (SBP), respiratory rate (RR), and peripheral oxygen saturation (SpO_2_) were continuously monitored throughout the study period and any adverse changes in these parameters were noted down for further analysis. An adverse respiratory event was defined as SpO_2_ < 92% and/or RR < 8 breaths/min. A decrease in SpO_2_ to less than 92% for more than 30 s was treated sequentially with verbal stimulation, head tilt-chin lift, Guedel airway, and bag-mask-assisted ventilation. RR < 8 breaths/min was treated sequentially with verbal stimulation, mild prodding, and nasopharyngeal stimulation. Hypotension was defined as a fall in SBP by >20%. A persistent (two readings 2 min apart) or recurrent [≥2 times during the study period (BL to PP60)] SBP < 90 mmHg was treated with boluses of IV ephedrine 6 mg. Hypertension was defined as a persistent or recurrent rise in SBP by >20% and was treated with boluses of IV labetalol (5 mg increments). Bradycardia was defined as an absolute decrease in HR < 55 beats/min. Persistent (more than 30 seconds) or recurrent bradycardia was treated with IV atropine 0.6 mg. Tachycardia was defined as a persistent rise in HR by >30% above baseline and was treated with additional sedation and analgesia. The physician performing the CVCI procedure is responsible for data collection and analysis.

The power of this study was calculated based on previous reported pain scores of 67 mm (standard deviation 25 mm) on 100 mm VAS during subcutaneous injection of LA in healthy volunteers [8]. A reduction in pain scores of 25 mm was considered acceptable to detect a clinically significant improvement [9]. Seventeen patients were required in each group, for an alpha-error of 0.05 and beta-error of 0.20. Therefore, sixty consecutive patients meeting the inclusion criteria were allocated randomly to one of three groups using a computer-generated random number list with sealed opaque envelope technique.

Statistical analyses were performed using software IBM Corp (2011), IBM SPSS statistics for windows, version 20.0 (Armonk, NY). Approximate normality of the distribution was assessed using Shapiro-Wilk test before applying a particular statistical test. Patients' characteristics and baseline continuous variables were compared using one-way ANOVA followed by Bonferroni* post hoc* test. Categorical data were analyzed using proportion chi-square test. A value of *P* < 0.05 was considered to be statistically significant. Pain, discomfort, and sedation scores were compared among the groups using Kruskal-Wallis test. Follow-up tests were conducted to evaluate pairwise differences among the three groups, controlling for Type I error across tests by using the Bonferroni approach (*P* < 0.016).

## 3. Results and Discussion

**Figure 1:**
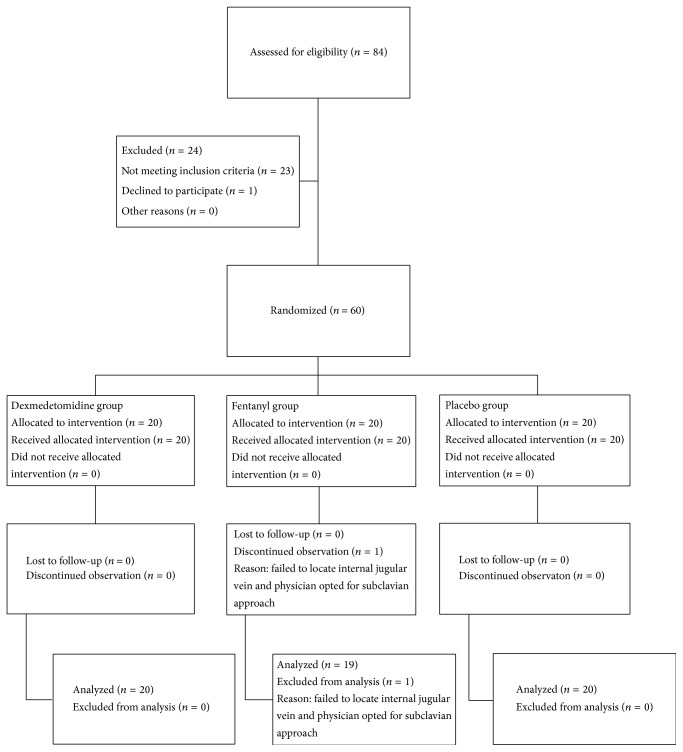
CONSORT patient flow chart.

Sixty patients were randomized, with all but one completing the study. One patient from fentanyl group was withdrawn due to failure in identifying a patent jugular vein and the physician opted for cannulating the subclavian vein (Figure 1, CONSORT flow diagram).

There were no significant differences among the three randomized groups of patients in terms of patient demographics, baseline respiratory, cardiovascular parameters, and number attempts for a successful CVCI (Table 1).

**Figure 2:**
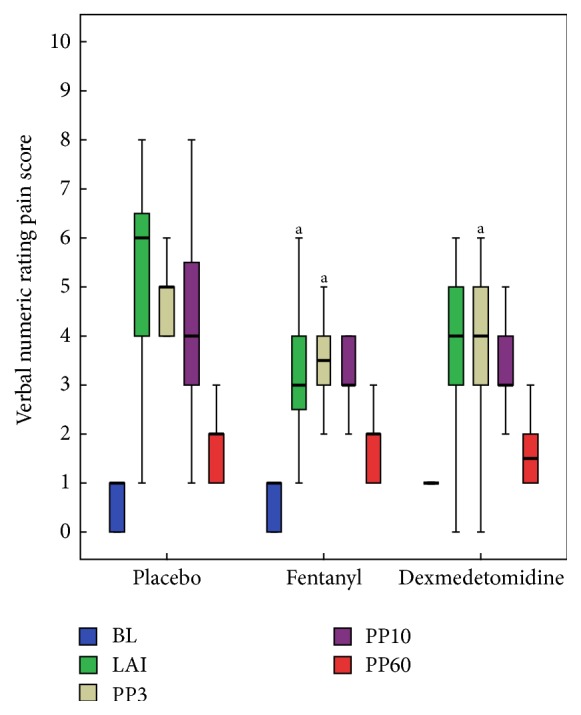
Box plots of perceived pain score during the study period showing median and interquartile range (25–75). BL, before starting study drug infusion; LAI, after initial LA injection; PP3, immediately after the CVCI, the patient was asked to report the peak pain experienced during the procedure; PP10, 10 min after completion of the procedure; and PP60, 60 min after completion of the procedure. ^a^ *P* < 0.016 versus placebo.

**Figure 3:**
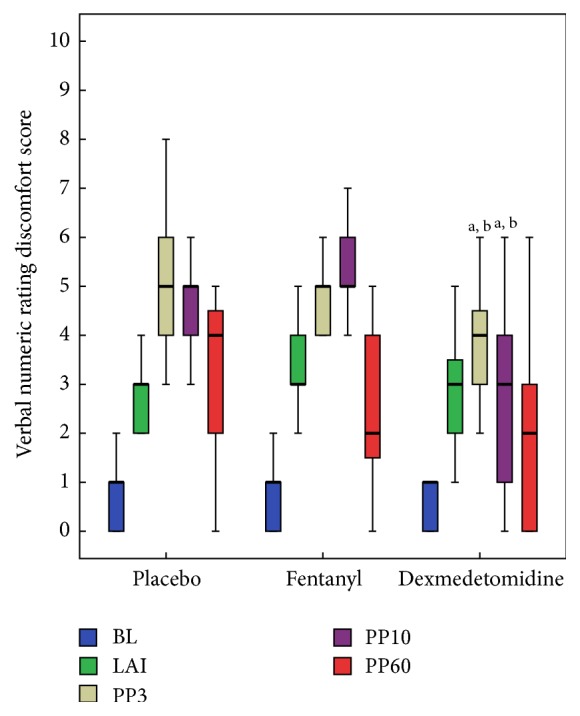
Box plots of perceived discomfort score during the study period showing median and interquartile range (25–75). BL, before starting study drug infusion; LAI, after initial LA injection; PP3, immediately after the CVCI, the patient was asked to report the peak pain experienced during the procedure; PP10, 10 min after completion of the procedure; and PP60, 60 min after completion of the procedure. ^a^ *P* < 0.016 versus placebo; ^b^ *P* < 0.016 versus fentanyl.

The median pain, discomfort, and sedation scores in dexmedetomidine, fentanyl, and placebo groups are presented in Figures 2 and 3 and Table 2, respectively. Comparison among the groups revealed that addition of fentanyl [3 (2–4)] and dexmedetomidine [4 (3–5)] attenuated the pain response to local anaesthetic injection in comparison to placebo [6 (4–6.7)] but it reached statistical significance only with fentanyl [*P* = 0.002; 95% CI, fentanyl versus placebo, −3.30 to −0.68]. However, in the immediate postprocedural period (PP3), both the study drugs attenuated the pain response and reached statistical significance in comparison to placebo [95% CI, dexmedetomidine versus placebo, −2.35 to −0.05, and 95% CI, fentanyl versus placebo, −2.81 to −0.47]. In contrast to dexmedetomidine, fentanyl appeared to be more analgesically efficient in reducing the pain intensity numerically to CVCI after LA injection (LAI) but it has not reached statistical significance [LAI, *P* = 0.002;  95% CI, dexmedetomidine versus fentanyl, −0.72 to 1.90]. The median VNRPS thereafter (at PP10 and PP60) were comparable among three groups (Figure 2). However, the procedure related discomfort was significantly lower in dexmedetomidine compared to fentanyl and placebo at each step of the procedure (PP3 and PP10) after LA injection (Figure 3).

Dexmedetomidine group had marginally significant higher incidences of bradycardia (*P* = 0.049) and hypotension (*P* = 0.046) than fentanyl and placebo groups. During study period, the absolute heart rate decreased below 55 in five, two, and nil patients from dexmedetomidine, fentanyl, and placebo groups, respectively. Two patients from dexmedetomidine group experienced bradycardia requiring treatment with atropine (0.6 mg bolus intravenous). Hypotension was evident in more number of patients from dexmedetomidine group (3/20) in contrast to fentanyl (0/19) and placebo (0/20) groups but did not require any treatment. One patient from fentanyl group experienced episodes of SpO_2_ < 92% and nausea compared to none from dexmedetomidine and placebo groups. However, the difference did not reach statistical significance. No patient vomited or required antiemetic medication (Table 2). None of our patients exhibited persistent tachycardia or hypertension needing intervention.

The median sedation score for dexmedetomidine group was significantly less (patients are more sedated) compared to placebo at LA injection and for the subsequent procedural steps. We did not find any significant difference in median sedation score between fentanyl and placebo groups. However, dexmedetomidine group patients were significantly more sedated compared to placebo and fentanyl groups at the end of the procedure (PP3). At the end of study period (PP60) all the patients were responding to verbally spoken words (OAA/S score of 4 or 5). No patient from any group manifested uncontrolled movement strong enough to hinder performance of the procedure (Table 2).

Overall, fentanyl and dexmedetomidine groups were not statistically different with median pain score at any time points, though fentanyl group patients trended towards less pain in the postprocedural period (LAI, PP3, and PP10). However, dexmedetomidine group patients had a distinctly better discomfort score in the postprocedural period (PP3 and PP10).

## 4. Discussion

Traditionally pain has been described as a combination of unpleasant sensory and emotional component. In our study, the sensory and emotional components of pain that might have been resulted from CVCI were measured with VNRPS and VNRDS, respectively.

The primary findings of the study are that subcutaneous LA infiltration induces maximum pain in the placebo group and preprocedural fentanyl and dexmedetomidine reduced this pain response. However, in terms of patients' comfort dexmedetomidine appears to be more efficient than fentanyl in the immediate postprocedural period.

Two American studies have described pain and discomfort as two separate perceptions experienced by patients during CVCI [6, 10]. The later study in their five-point numeric rating scale described CVCI as a severely uncomfortable and moderately painful procedure [10].

Fentanyl has been studied extensively for pain management and its efficacy is well documented for management of acute pain [11, 12]. However, the reason for choosing dexmedetomidine is its additional hypnotic, sedative, and anxiolytic properties with minimal respiratory depression [13, 14].

Short acting opioid has been favored for brief painful procedures. An Indian study concluded that both fentanyl and sufentanil boluses 10 min before chest tube removal reduce the mean pain intensity score significantly compared to placebo control at 5 and 20 min in the recovery room [15]. Similar evidence comes from an Irish study which used three different rates of remifentanil infusion along with LA field infiltration and propofol sedation for long term insertion or removal of central venous access devices [16]. However, most of the patients in their allocated study group required a bolus of rescue analgesic indicating that different procedural steps of CVCI require different concentration of remifentanil. In our study, fentanyl is more analgesically efficient compared to dexmedetomidine for the first step of CVCI (LA injection). However, for the second procedural step (PP3, at the end of procedures) both fentanyl and dexmedetomidine are equally analgesically efficient compared to placebo, though fentanyl still appears to be more analgesically efficient than dexmedetomidine with lower VNRPS. This implies that appropriately timed preprocedural bolus infusion of fentanyl and dexmedetomidine is a simple alternative to continuous infusion in nontunneling, short duration CVCI. Our procedures did not require tunneling and were of short duration so we have not used continuous infusion in our study.

The sole use of dexmedetomidine as a sedative analgesic is not consistently successful for many moderately painful invasive procedures. A multicenter American study suggested that the surge in pain arising out of CVCI in a conscious patient may lead to central sensitization and could be a potential cause for persistent pain which continues for some time after a noxious event and advocated use of preemptive analgesia to avoid central sensitization [2]. In the current study, baseline analgesia was provided with preprocedural infusion of either dexmedetomidine or fentanyl and both the study drugs were able to reduce procedure specific pain at LA injection and immediately after CVCI (PP3) in comparison to placebo group (*P* = 0.001 and 0.003). However, fentanyl appeared to be more analgesically efficient than dexmedetomidine in reducing the pain intensity at all time points (LAI, PP3, and PP10) but it has not reached statistical significance. In contrast, there is a sharp statistical difference in discomfort level as assessed by VNRDS between fentanyl and dexmedetomidine groups indicating superiority of dexmedetomidine over fentanyl in the immediate postprocedural period (PP33 and PP10). This difference in action between fentanyl and dexmedetomidine can be partly explained by multidimensional model of procedural pain [6]. Fentanyl, which acts as a pure opioid analgesic, mainly affected the sensory-discriminative component of the pain, whereas dexmedetomidine had a greater magnitude of effect in attenuating the motivational affective and cognitive component of pain. The differential action can further be explained by the fact that both the drugs have different timing for onset of action, peak onset of action, and terminal elimination half-life after short bolus infusion over 10 minutes.

All patients from dexmedetomidine group had peripheral oxygen saturation of more than or equal to 99% by pulse oximeter in room air. However, more number of patients from dexmedetomidine group also attained an unwanted sedation level (lower OAA/S sedation score) during the study period which may sometime hinder the patient cooperation needed by the physician while inserting central venous catheter. This finding also confers the advantage of dexmedetomidine in providing adequate sedation with clinically insignificant respiratory effects, including apnea, hypoxemia, or airway obstruction during ICU procedures. However, a higher incidence of bradycardia and hypotension associated with dexmedetomidine is a worrisome predicament. A previous study using a higher dose of fentanyl (2 *μ*g/kg) reported higher incidence (15%) of oxygen desaturation [3]. We have used a single preprocedural bolus fentanyl 1 *μ*g/kg infusion and reduced the risk of clinically significant respiratory depression requiring intervention like head tilt-chin lift maneuver, naloxone administration, and resuscitation with bag-mask ventilation, without compromising the analgesic efficacy. Only one patient from fentanyl group in our study desaturated (SpO_2_ < 92%) and needed a simple sequential verbal stimulation to maintain peripheral oxygen saturation above 98%.

The major limitation of the current study cohort is a possible risk of inclusion bias with limited external validity as the decision to place a central venous catheter was at the discretion of attending anesthesiologist. Many patients who will need a CVCI may not belong to American Society of Anesthesiologists physical status grade 1/2 with age around 40 years; even then we feel this study might serve as a pilot for further research in more critically ill patients. The second limitation is a precision bias owing to small sample size. All the study participants were premedicated with a dose of anxiolytic on the night before surgery and also on the morning of surgery which might have influenced the motivational components of pain and the effect size due to possible potentiating effect of the study drugs, but since all included subjects were given the same premedication, this may not really represent a major limitation. The third limitation is the fact that unavailability of an ultrasound for CVCI and lack of the ultrasonographic assistance could have been a confounding factor as it could have reduced the procedure time and number of attempts for CVCI, both of which might correlate to patient's sense of discomfort and pain. However, we are unaware of any study comparing the pain and discomfort associated with ultrasound guided CVCI versus conventional technique of CVCI.

## 5. Conclusions

In conclusion, both fentanyl and dexmedetomidine provide comparable analgesia for CVCI along with LA field infiltration. Dexmedetomidine is superior to fentanyl and placebo in terms of providing comfort to the patients during the procedure but is associated with a tendency to excessive sedation and unwanted cardiovascular events. With minimal adverse respiratory and cardiovascular events and a comparable analgesic efficacy, fentanyl 1 *μ*g/kg is a better alternative to provide additional analgesia for CVCI. Further studies are required to see whether reduced preprocedural dexmedetomidine infusion will bring a beneficial cardiovascular and respiratory effect without compromising analgesia and patient's comfort level.

## Figures and Tables

**Table 1:** Patients characteristics and baseline variables.

Variable	Dexmedetomidine (*n* = 20)	Fentanyl (*n* = 19)	Placebo (*n* = 20)	*P* value
Age in years	39.4 ± 13.5	38.4 ± 11.4	40 ± 9.7	0.898
M/F (*n*)	13/7	12/7	9/11	0.370
Weight (kg)	56 ± 11.9	56 ± 12.5	59 ± 12.3	0.703
ASA PS^a^ 1/2 (*n*)	12/8	13/6	14/6	0.774
Heart rate	81 ± 8.5	80.5 ± 7.2	82 ± 8.5	0.830
SBP (mmHg)^b^	126.4 ± 14.9	125 ± 11.2	125.5 ± 13.8	0.967
SpO_2_ (%)	99.6 ± 0.5	99.7 ± 0.4	99.6 ± 0.6	0.738
Respiratory rate	17.7 ± 1.8	17.4 ± 1.8	17.2 ± 2.0	0.664
Number of attempts^c^	1.1 ± 0.36	1.2 ± 0.45	1.2 ± 0.41	0.692
Procedural time^d^ (in seconds)	1423 ± 206	1261 ± 232	1304 ± 275	0.100

Values are mean ± standard deviation or numbers (*n*).

^a^American Society of Anesthesiologists physical status 1/2.

^b^SBP: systolic blood pressure.

^c^An attempt is defined as the introducer needle's entry into the skin and its removal from the skin.

^d^Time from skin puncture to anchoring the last suture to the skin for central venous catheter fixation.

**Table 2:** Adverse outcomes and sedation score during the study period.

Adverse events	Dexmedetomidine (*n* = 20)	Fentanyl (*n* = 19)	Placebo (*n* = 20)	*P* values
Hypotension (*n*)	3	0	0	0.046
Bradycardia (*n*)	5	2	0	0.049
Desaturation (*n*)	0	1	0	0.343
Nausea (*n*)	0	1	0	0.343
Observer's assessment of alertness/sedation score, median (interquartile range 25–75).				
BL	5 (5-5)	5 (5-5)	5 (5-5)	1.000
LAI	4 (3–5)^a^	4 (3–5)^a^	5 (5-5)	0.001
PP3	4 (3–5)^a^	5 (4-5)	5 (5-5)	0.001
PP10	4 (3-4)^a,b^	5 (5-5)	5 (5-5)	0.000
PP60	5 (4-5)	5 (5-5)	5 (5-5)	0.060

*n*: number of patients.

^a^ *P* < 0.016 versus placebo; ^b^ *P* < 0.016 versus fentanyl.

Observer's Assessment of Alertness/Sedation Scale: 0, does not respond to deep stimulus; 1, does not respond to mild prodding or shaking; 2, responds only after mild prodding or shaking; 3, responds only after name is called loudly and/or repeatedly; 4, lethargic response to name spoken in normal tone; 5, responds readily to name spoken in normal tone (alert); 6, agitated.

BL, before starting study drug infusion; LAI, after initial LA injection; PP3, immediately after the CVCI, the patient was asked to report the peak pain experienced during the procedure; PP10, 10 min after completion of the procedure; and PP60, 60 min after completion of the procedure.
